# Pre-Clinical Drug Prioritization via Prognosis-Guided Genetic Interaction Networks

**DOI:** 10.1371/journal.pone.0013937

**Published:** 2010-11-10

**Authors:** Jianghui Xiong, Juan Liu, Simon Rayner, Ze Tian, Yinghui Li, Shanguang Chen

**Affiliations:** 1 School of Computer Science, Wuhan University, Wuhan, People's Republic of China; 2 Bioinformatics, Systems Biology and Translational Medicine Group, State Key Lab of Space Medicine Fundamentals and Application, China Astronaut Research and Training Center, Beijing, People's Republic of China; 3 Bioinformatics Group, State Key Laboratory of Virology, Wuhan Institute of Virology, Chinese Academy of Sciences, Wuhan, People's Republic of China; 4 Department of Medical Oncology, Dana-Farber Cancer Institute, Harvard Medical School, Boston, Massachusetts, United States of America; Lund University, Sweden

## Abstract

The high rates of failure in oncology drug clinical trials highlight the problems of using pre-clinical data to predict the clinical effects of drugs. Patient population heterogeneity and unpredictable physiology complicate pre-clinical cancer modeling efforts. We hypothesize that gene networks associated with cancer outcome in heterogeneous patient populations could serve as a reference for identifying drug effects. Here we propose a novel *in vivo* genetic interaction which we call ‘synergistic outcome determination’ (SOD), a concept similar to ‘Synthetic Lethality’. SOD is defined as the synergy of a gene pair with respect to cancer patients' outcome, whose correlation with outcome is due to cooperative, rather than independent, contributions of genes. The method combines microarray gene expression data with cancer prognostic information to identify synergistic gene-gene interactions that are then used to construct interaction networks based on gene modules (a group of genes which share similar function). In this way, we identified a cluster of important epigenetically regulated gene modules. By projecting drug sensitivity-associated genes on to the cancer-specific inter-module network, we defined a perturbation index for each drug based upon its characteristic perturbation pattern on the inter-module network. Finally, by calculating this index for compounds in the NCI Standard Agent Database, we significantly discriminated successful drugs from a broad set of test compounds, and further revealed the mechanisms of drug combinations. Thus, prognosis-guided synergistic gene-gene interaction networks could serve as an efficient *in silico* tool for pre-clinical drug prioritization and rational design of combinatorial therapies.

## Introduction

The development of effective cancer drugs is a particularly challenging problem, and selection of appropriate preclinical cancer models has emerged as a key factor affecting successful oncology drug discovery and development [1]. There are multiple examples of drug candidates that showed promise in the pre-clinical stage but which then failed to demonstrate benefits in clinical trials. EGFR- and VEGF-blocking combo are recent examples of drugs which ultimately produced disappointing results after encouraging pre-clinical results [2]. One of the commonly accepted reasons is that the targeted therapies provide benefit only to a subset of patients who have the appropriate genetic changes in their cells; for example, Herceptin (trastuzumab) shows efficacy only in HER2-positive breast cancers [3]. Thus the key to success in the clinical stage may depend strongly on precise selection of target populations.

In the modern drug discovery pipeline, assessments of the efficacy and toxicity of therapeutic agents are based on relatively homogeneous cell or animal models, and the heterogeneity issue is only encountered once the most expensive clinical trials are underway in human subjects. The poor success rate of oncology drug development suggests that the standard preclinical cancer models are failing to predict how the drug candidate works in clinical trials [4]. Furthermore, recent results from comprehensive genomic efforts such as The Cancer Genome Atlas (TCGA) have highlighted the marked heterogeneity of genetic alterations in patient populations [5]. It suggests that the intrinsic heterogeneity in genetic and/or epigenetic alterations which are driving the tumorigenesis might be one of the main causes for the observed discrepancies between clinical trials and standard pre-clinical models. Thus, efforts to establish new cancer animal models which mimic heterogeneous patient populations might be even more challenging than initially realized [1], [4].

Nevertheless, several promising new paradigms in cancer drug development have recently been introduced of which Network Pharmacology and Synthetic Lethality seem to hold particular promise. Network Pharmacology attempts to model the effects of a drug action by simultaneously modulating multiple proteins in a network [6], [7]. However, this approach still faces a number of challenges. In particular, the absence of cancer-specific functional gene/protein networks and the lack of further characterization of the network behavior (e.g, network robustness [8] under perturbation) makes it difficult to design an accurate perturbation strategy [6], [9]. Synthetic Lethality refers to a specific type of genetic interaction between two genes, where mutation of one gene is viable but mutation of both leads to death [10]. It has already been demonstrated that this concept can be exploited to develop a therapeutic strategy. For example, by using an inhibitor targeted to a Poly(ADP-Ribose) Polymerase (PARP) that is synthetically lethal to a cancer-specific mutation (BRCA), researchers could target cancer cells to achieve antitumor activity in tumors with the BRCA mutation[11]. However, because of the difficulties of systematically identifying in vivo synthetic lethal genes in human individuals, current high throughput Synthetic Lethality screening is limited to only in vitro cell lines [12].

Transcriptome profiles of heterogeneous patient populations have been comprehensively sampled by high throughput gene expression microarrays in ongoing prognosis studies (the original motivation being to identify gene expression signatures for prognostic or predictive biomarkers) [13]. Recognizing this, we propose that this kind of patient prognosis data could be used to help prioritize drug candidates or drug combinations at the pre-clinical stage. To test the feasibility of this hypothesis, we combined microarray gene expression data with cancer prognostic information to identify cancer-specific gene-gene interactions. We achieved this by defining a set of ‘gene modules’ and then used the microarray data to identify cancer specific gene interactions that occurred between genes in different modules. A single gene module represented a list (as opposed to a network) of genes that shared a similar function or regulatory mechanism and was defined as one of the following four collections: (1) a group of genes in a protein-protein interaction network or protein complex; (2) a set of genes sharing a common function annotation in the Gene Ontology; (3) a set of genes which are involved in the same pathway; (4) a set of genes which are governed by a common regulation mechanism. i.e., targets of the same microRNA. The gene interactions were identified by using an information theoretic measure of synergy[14] based on the microarray expression data. Two genes that are identified to be synergistically related form a “Synergistically Inferred Nexus” (SIN). These SINs together form an inter module network where the nodes in the constructed network represent functional gene modules, and links between two nodes represent interactions between modules. We found that the constructed network contained a number of highly connected nodes and, given the potential pivotal role of the associated modules in affecting patient outcome, we named them *‘gatekeeper’* modules. Furthermore, by examining their associated GO terms, we found that *drug accessibility, microenvironment and immune system regulation* are common themes in the *gatekeeper* modules identified from multiple types of human cancers.

Finally, by projecting drug sensitivity-associated genes on to the cancer-specific inter-module network, we defined a ‘perturbation index’ to quantify the potential efficacy of drugs in terms of the drugs' perturbation pattern on the inter-module network (see **Methods**). We demonstrated that this index could successfully discriminate drugs from candidate pools (i.e., drug candidates in the NCI Standard Agent Database, see **Methods**). With this approach, we have illustrated an objective way to quantify the synergistic effects of drug combinations, and the rationale of combinatorial perturbations on these intrinsic cooperation networks. Thus, the integration of action data (describing the effect of a drug acting on a cell) with an intrinsic gene network (derived from a patient population) not only provides a novel *in silico* prioritization tool in the early preclinical stage, but can also suggest a potential treatment strategy based on the gene networks.

## Results

### The framework of *in silico* modeling

**Figure 1:**
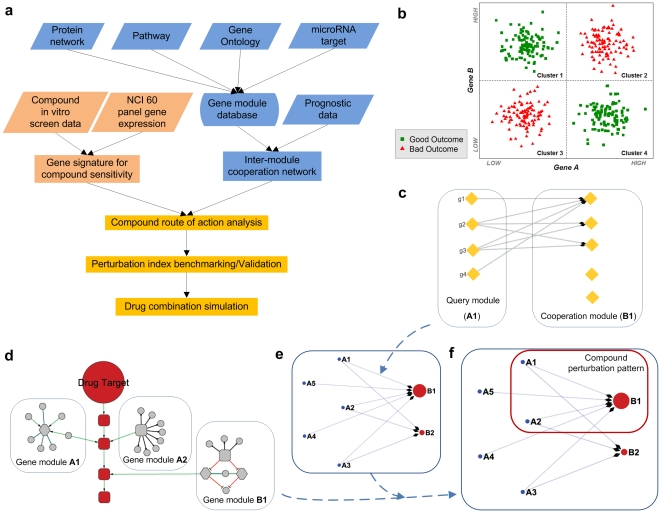
The proposed schema for compound Pattern of Action (POA) analysis. **a**. The workflow of POA analysis, which relies on converging two lines of information: the intrinsic module structure which cooperatively determine the clinical prognosis outcome of heterogeneous patient population (blue rectangles); and the gene signatures for compound sensitivity resulting from in-vitro cell line screen (pink rectangles). **b**. Illustration of the ‘synergistic outcome determination’ (SOD), a proposed in vivo gene-gene functional interaction. SOD is defined as the synergy of a gene pair with respect to cancer patients' outcome. Here gene A and gene B have two states: high expression or low expression level. Red triangles represent ‘bad outcome’ patients (shorter survival time or metastasis), and green rectangles represent ‘good outcome’ patients (longer survival time or non metastasis). In combination, the two genes are sufficient to determine the patient outcome, but each of them individually is uncorrelated with patient outcome. For example, given gene A state as ‘low expression’, all patients with A(Low) are distributed in two clusters and thus insufficient to determine the patients outcome. Given combination of A and B state, i.e., A(Low) B(high), its sufficient to determine the patient outcome as ‘good outcome’. **c**. Inter-module cooperation network construction. For each gene (*g1*∼*g4* at left) in a given gene module, we identify their synergistic partner genes (the link from gene in module A1 to gene module B1 form a ‘Synergistically Inferred Nexus’, see **Methods**). Then the gene modules which are over-represented in the resulting gene list are identified as the ‘cooperative modules’ corresponding to the query gene module. **d**. Compound perturbation pattern. Genes associated with compound sensitivity (nodes within blocks) might be topologically cross-linked to the functional pathway (red rectangles) induced by compound perturbation. **e**. Disease specific inter-module cooperation network, nodes represent gene modules and the direct link represents the relationship between the ‘query module’ (**A1**) and its ‘cooperative module’ (**B1**). Here **B1** cooperates with a large number of modules (with flow-in links), thus we called this special class of modules ‘gatekeeper modules’ (**B1**, **B2**) and others (without flow-in links) as ‘checkpoint modules’ (**A1–A5**). **f**. The Pattern of Action (POA) of one candidate compound generated by overlapping the disease-specific inter-module network (**e**) with the module hits by sensitivity-associated genes (**d**).

The basic framework of our modeling method is illustrated in **Figure 1a**. There are three independent components in the method: (1) construction of gene modules; (2) identification of disease-specific gene-gene interactions from patient gene expression and prognosis data; (3) identification of drug sensitivity associated genes.

**Figure 2:**
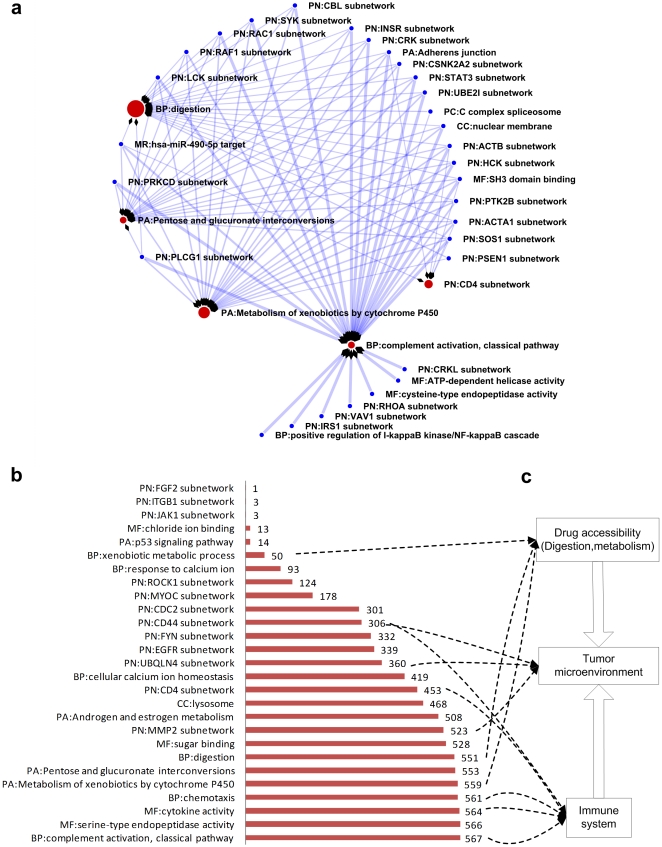
Topological characteristics of the Pattern of Action network. **a**. Gatekeeper modules and checkpoint modules, demonstrated by an example (the POA result of Cisplatin on non-small cell lung carcinoma). We define the flow-out nodes (blue circles) as ‘checkpoint modules’ (from gene signatures of drug sensitivity), and the flow-in nodes (red circles) as ‘gatekeeper modules’ (which cooperate with a large number of modules to determine the clinical prognosis outcome). The radius of the red circles is proportional to the in-degree (number of flow-in links) of the node in the generic inter-module cooperation network. **b**. All of the ‘gatekeeper’ modules in a generic inter-module cooperation network generated for lung cancer (non-small cell lung carcinoma). The length of bars and annotated numbers indicate in-degree (number of flow-in links) for each gatekeeper module (y-axis). **c**. An ensemble of common gatekeeper modules in multiple cancer types highlights a physiology-level ‘pathway’ of drug action. Gene module names start with a 2-character header that indicates the gene module definition source, PN: protein subnetwork; PA: pathway; BP: Gene Ontology biological process; MF: Gene Ontology molecular function; CC: Gene Ontology cellular component; MR: microRNA targets.

A key step in the method is the identification of gene-gene functional interactions as synergistic events; these events are determined not only by gene expression data but also by prognosis. The proposed in vivo genetic interaction which we call ‘synergistic outcome determination’ (SOD) is a concept similar to ‘Synthetic Lethality’ [10]. SOD is defined as the synergy of a gene pair with respect to cancer patients' outcome, whose correlation with outcome is due to cooperative, rather than independent, contributions of genes (see **Methods**). Identification of a synergistic gene pair leads to the creation of a Synergistically Inferred Nexus (SIN) which, when combined with other SINs, produces an Inter-Module Cooperation Network (IMCN). An important distinction between our method and the concept of Synthetic Lethality is that in the latter the phenotype is defined at the cell-level (i.e. cell death), whereas we define the phenotype at the physiological level (i.e. the survival outcome of the individual). Furthermore, the gene expression profiling data for a tumor is from a mixture of tissues which include epithelial cells and other cells in the microenvironment; thus a SIN captures events at the tissue level rather than at the cell level. This also leads to differences in the interpretation of the constructed network. In Synthetic Lethality, the nodes represent individual genes, but we use a gene module as the principal unit and thus capture a higher level inter-module of cooperation. We mapped a list of genes onto a set of gene modules according to a comprehensive range of functional data based on currently available sources (the gene function annotation database, protein network and protein complexes, annotated pathways, and genes co-regulated by microRNA, **Figure 1a** and **Methods**). The reasons for capturing module level cooperation rather than considering the interactions between individual genes were as follows: (1) a gene module (or corresponding ‘gene set’) is a more appropriate representation of the functionality of the system, which occurs as a series of interactions between elements. It is widely accepted that one shortcoming of microarray prognosis experiments is the low reproducibility. It often leads to completely different prognosis-associated gene signatures based on different patient cohorts. Considering that the subnetwork marker extracted from protein interaction databases are more reproducible than individual gene markers [15], [16], we assume that the identification of module-module interactions is more robust than that of gene-gene interactions. For example, if the interaction between gene modules A1 and B1 (in **Figure 1c**) is true, then many of the genes in gene module A1 could interact with a many of the genes in module B1. The robust identification of individual gene-gene interactions between A1 and B1 is harder, because it is possible that different set of B1 genes will be identified as interacting with A1 genes when different patient cohorts or microarray datasets are examined (**Figure 1c**); (2) Multiple genes within a gene module might have redundant functionality, and a tumor could exploit alternative pathways or mechanisms within a gene module to develop drug resistance [17], [18]. Since therapeutic intervention targeting different yet functionally redundant genes within a gene module might be equivalent, it is important to highlight a drug perturbation pattern on an inter-module rather than an intra-module network.

There are two methods which are commonly used to interrogate the action of compounds on cells. The first method, adopted by the Connectivity Map [19] effort, measures a ‘compound response signature.’ In this approach gene expression signatures are established based on changes in gene expression in response to short term treatment with particular compounds; this response signature can serve as an effective tool for probing the compound(s) mechanism of action (MOA) [19]. A second method is measurement of a ‘drug sensitivity signature’ and is used by various applications based on the National Cancer Institute NCI 60 in vitro drug screen project [20]. The NCI 60 cell lines screen panel has proved to be an effective way to identify drug sensitivity specific biomarkers [21] as the panel has already been comprehensively characterized via profiling at different levels (mRNA, protein, microRNAs, DNA methylation and metabolites etc.).

To incorporate the data describing the perturbation effects of drug compounds we constructed cancer specific inter-module cooperation networks based around the identified gene modules and using a query-based approach that incorporated both microarray gene expression data and prognosis information (**Figure 1e** and **Methods**). This inter-module network allowed us to mine the drug action pattern by incorporating drug-gene relationships. To represent the characteristic pattern of drug action on cells, we chose the genes that were significantly associated with drug sensitivity across NCI 60 cell lines (drug-gene association [21], see **Methods**). As illustrated in **Figure 1d**, although these genes may not be directly linked to the primary drug targets (i.e. the mechanism of action), they should be close to the pathway in which the targets are involved. Thus these drug sensitivity associated genes can indicate the key pathways associated with drug efficacy [21] and the overlap of sensitivity associated genes (**Fig. 1d**) with the baseline inter-module network (**Fig. 1e**) could highlight the characteristic compound perturbation pattern, which we call the Pattern of Action (POA, **Fig. 1f**). For simplicity, the genes which were significantly correlated with compound(s) sensitivity across 60 cell lines were selected (**Methods**) and are referred to as compound gene ‘hits’ in this report.

### Inter-module networks associated with prognosis outcome

If two genes A and B could synergistically determine or predict prognosis outcome (form a SIN), we call B a synergistic partner of A (or vice versa). By enumerating all genes in a gene module and identifying their synergistic partners, followed by further identifying the enriched gene modules within these partners (**Supporting Text S1**), the inter-module network was first constructed for a patient population of non-small cell lung carcinoma (NSCLC), a major type of lung cancer. To get a more specific view of the constructed networks, we illustrate a network hit by Cisplatin (the first line treatment option for NSCLC) in **Figure 2a**.

Analysis of the inter-modules network obtained from three other types of cancer (breast cancer, ovarian cancer and leukemia, see **Supporting Text S1**), also identified common features shared between these networks. Specifically, there exist several hub nodes which have a large number of flow-in links, indicating they play a central role in determining the clinical outcome. We named these highly connected influx nodes ‘gatekeeper modules’ and other outflux nodes as ‘checkpoint modules’.

**Figure 2b** illustrates the high connectivity of the gatekeeper modules. In the intrinsic network for lung cancer (NSCLC), a small set of gatekeeper modules cooperate with a large number of modules (in terms of outcome prediction). The largest identified hub was ‘BP: complement activation, classic pathway’ which, according to the Gene Ontology biological process definition, has cooperation with 567 checkpoint modules. This high connectivity was evident in all the four types of cancers we studied as there was significant overlap amongst most of the gatekeeper modules (see **Figure S1** for gatekeeper modules of breast cancer, ovarian cancer and leukemia).

Based on this analysis, the biological themes of the most highly connected gatekeeper modules in multiple types of cancer are summarized in **Figure 2c**, and comprise 3 major themes: (1) drug accessibility to tumor cells (drug absorption/metabolism/delivery), (2) tumor microenvironment and (3) immune regulation (also a key component of the tumor microenvironment). These common themes indicate the pivotal role of the *in vivo* tumor microenvironment, and the efficacy of drugs could be regulated by these components (**Figure 2c**). For example, the control of drug accessibility to tumor cells by increasing the efflux of the drug molecules (multidrug resistance) is a major factor in the failure of multiple forms of chemotherapy [22]. Furthermore, the most common gatekeeper module identified is ‘BP: complement activation, classic pathway’, which plays a pivotal role in the ‘fine tuning’ of both the innate and cognate immune responses [23]; there is evidence that shows a tumor could exploit the complement activation to set up an immunosuppressive microenvironment, thereby gaining a growth advantage [24].

Considering the increased recognition of the complexity of tumor regulation *in vivo*, the difficulty of identifying effective cancer cures (as evidenced by drug resistance) may be a consequence of the robustness of physiology-level (or microenvironment-level) network regulation [8]. Our results suggest characterization of this cooperation network and the potential co-opt strategies which the tumor may exploit will aid in the development of new strategies to efficiently disrupt the highly robust network established by the tumor.

### Association of gatekeeper modules with genetic and epigenetic aberration events

**Figure 3:**
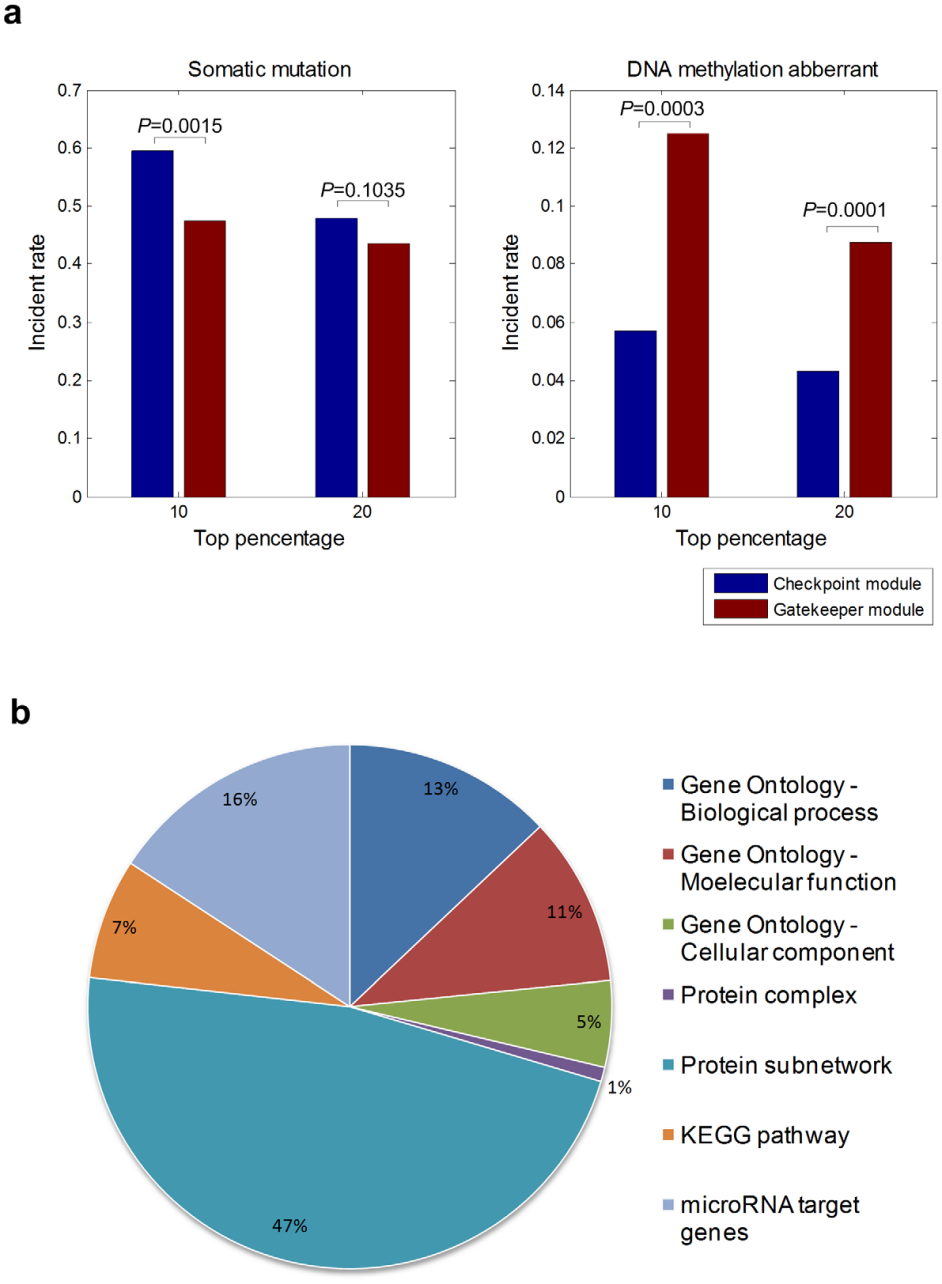
Biological function characterization of the inter-module cooperation network. **a**. For each type of module (gatekeeper and checkpoint), the top 10% and 20% most highly used genes are used as the representative genes for each module, and their incident rate of somatic mutation frequency and DNA methylation aberrant was calculated for lung cancer (NSCLC); p-value for incident rate difference was calculated using the binomial distribution (see **Methods**). **b**. Contribution of various evidence sources for gene module definition in lung cancer (NSCLC). We summarized the number of various types of gene module definitions in the identified inter-module network for lung cancer (NSCLC) and the proportional contribution of various evidence sources for the gene modules were plotted.

To characterize the intrinsic features of an inter-module network, particularly the identification of ‘gatekeeper modules’, we further compared the rates of genetic (somatic mutation) and epigenetic (DNA methylation) aberration on tumor vs. normal tissues. For each type of module, we selected genes which were identified as being highly used (i.e. one gene involved in multiple gene modules) as representative of the whole set (**Methods**). Results for the lung cancer (NSCLC) IMCN show that gatekeeper modules have a significantly lower incident rate of somatic gene mutation, but a notably higher incident rate of DNA methylation aberration (**Figure 3a**). All other types of cancers studied show a similar pattern (data not shown). Current strategies to treat cancer is mainly driven by identifying genetic changes (e.g., EGFR, epidermal growth factor mutations in lung cancer), but recent evidence suggests that epigenetic plasticity together with genetic lesions also drives tumor progression [25], [26]. Our data indicates most genes involved in gatekeeper modules frequently undergo epigenetic aberration during cancer, supporting the role of epigenetic lesions in tumor phenotype.

### Contribution of various evidence sources for gene module definition

Our gene modules were generated by integrating multiple large scale evidence of gene function categorizations such as protein-protein interaction networks, gene annotation databases, and microRNA target genes. To analyze the contribution from different evidence sources to the IMCN, we summarized the evidence sources in all gene modules of the lung cancer (*NSCLC*) network (**Figure 3b**). The other three types of cancers studied showed a similar pattern (data not shown). The top contribution was from protein-protein interaction subnetworks (47%) which were identified by simply fetching the neighboring proteins of hub nodes in a physical protein interaction network (**Methods**). Clearly, a more comprehensive decomposition of the modularity and community structures within a protein interaction network will provide a more extensive result set, given the large amounts of methodology and data from related systems biology studies [27]. It was not unexpected to see that the Gene Ontology, as a hierarchical knowledge representation system, made a major contribution to the definition of the gene modules (e.g. biological process category contributes 13%). However, it was more surprising to see that microRNAs modules made a similarly significant contribution of 16%, given these modules were defined by predicted microRNA target genes collected in the mirBase database [28].

### Perturbation index and validation

**Figure 4:**
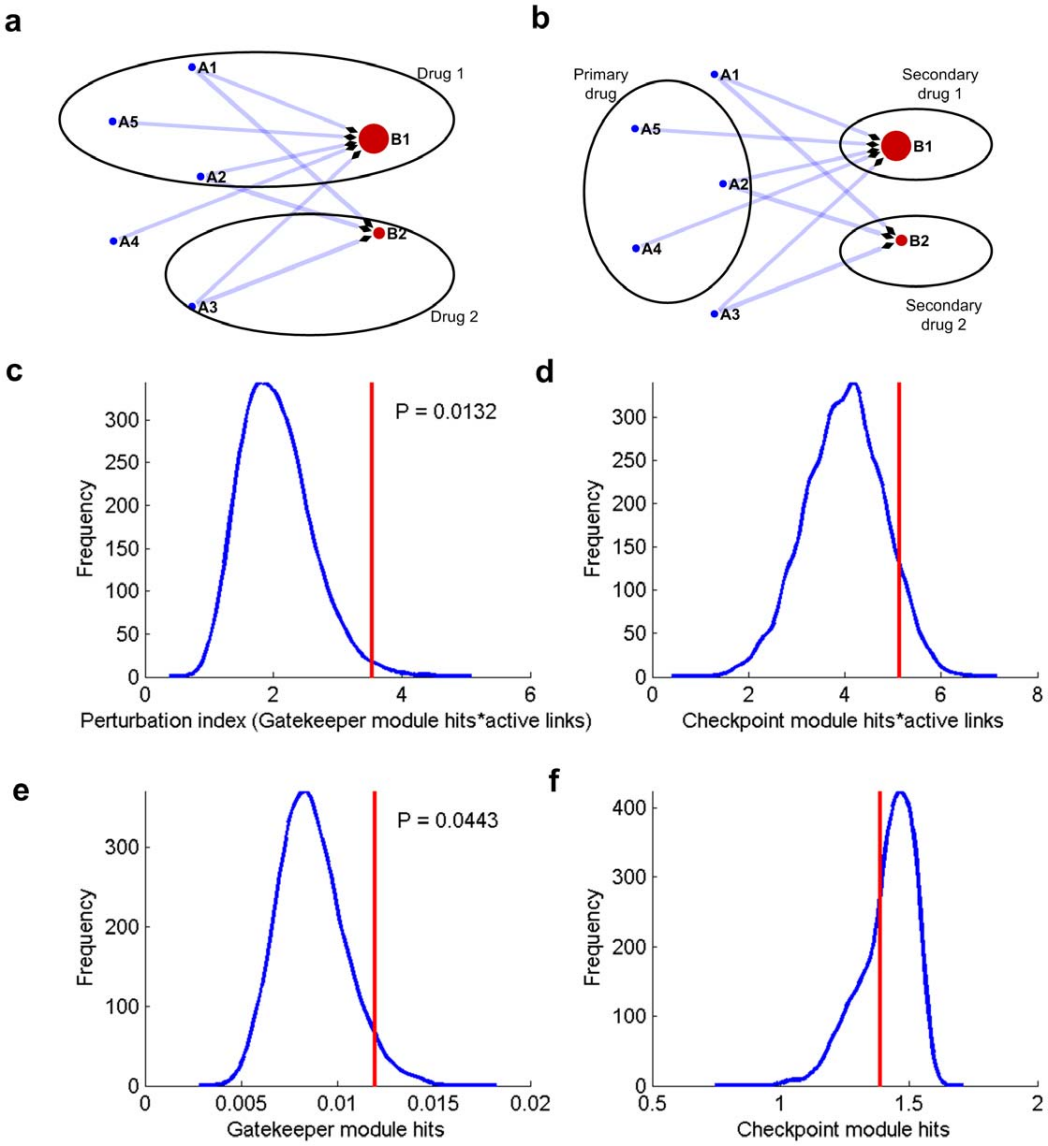
Principle and validation of perturbation index (*PI*). **a**. Perturbation index for single compound perturbation. According to our definition of *perturbation index* (***PI***, see **Methods**), ***PI*** (*drug 1*)  = 3 (Three active links from *A1, A2, A5* to *B1*), while ***PI*** (*drug 2*)  = 1 (one active link from *A3* to *B1*). **b**. The rationale of drug POA analysis applied to *in silico* drug combination assessment. If we assume one drug already has an established action (*primary drug* at left), then for each candidate auxiliary drug (shown at right), the perturbation index is re-calculated after adding the additional module hits provided by the secondary drug (see **Methods**). Here drug 1 is “better” than drug 2 because drug 1 has more active links (3 links from A5, A2 and A4) with the primary drug. **c**. Perturbation index can be used to discriminate successful drugs against candidate compounds. We use a bootstrap-based method to evaluate if the average ***PI*** of successful drugs against lung cancer (NSCLC) is significantly different from the candidate compounds (see **Methods**). Blue line shows background distribution and the red line shows the average ***PI*** of successful drugs. We also considered modified ***PI*** definitions and investigated their effect/contribution on the performance of ***PI***. These modifications include: **d**. bootstrap result from pseudo PI definition by using checkpoint modules information to replace gatekeeper modules information, **e**. bootstrap result from pseudo PI definition by only using gatekeeper modules hits, and **f**. bootstrap result from pseudo PI definition by only using checkpoint modules hits.

Based on the above characterization of the intrinsic features of the inter-module cooperation network, we hypothesized that the potential efficacy of drug intervention relies on its perturbation pattern on this network (**Figure 4a**). For a drug designed to perturb genetic aberrations (checkpoint modules), the key to success is whether it simultaneously perturbs the corresponding gatekeeper modules which cooperatively determine the outcome with the former. Thus there are two key factors in determining the extent of perturbation on the cooperation network: (1) the number of gene hits in gatekeeper modules and (2) the number of active links between gatekeeper modules and checkpoint modules (meaning simultaneous hits on gatekeeper modules and their linked checkpoint modules). As a measure of these quantities we defined the perturbation index (PI) as the summation of these two factors followed by appropriate normalization by the total number of gene hits (see **Methods**).

To assess the potential application of this approach for prioritizing compounds for clinical trials (based on the information available in pre-clinical stage), we studied a subset of compounds defined in the ‘*Standard Agent Database*’, originally created by Boyd [29] and ultimately finalized by the NCI. The selection criteria was compounds which have been submitted to the FDA for review as a New Drug Application, as well as compounds that have reached a particular high stage of interest at the NCI. For each type of cancer, we divided this compound list into two parts: FDA approved and routinely used drugs (the Successful drug list) and the remainder (the Candidate list), and tested whether we could statistically discriminate between these two compound lists using the perturbation index.

A bootstrapping-based method showed that the PI of successful compounds is significantly higher than the corresponding PIs for the candidate list in lung cancer (NSCLC) (p-value 0.01, **Figure 4c**). Because our perturbation index definition is highlighting the importance of gatekeeper modules, we also calculated a different measure of the perturbation index which is based on the number of gene hits in checkpoint rather than gatekeeper modules, multiplied by the number of active links, as a control. The result demonstrated that this modified index cannot achieve significant discrimination (**Figure 4d**), which confirmed the unique role of gatekeeper modules in drug efficacy. When we further removed the information contribution from active links and only counted the gatekeeper module hits, it turned out that there was a partial loss in discriminative power although the difference was still significant (p-value 0.04, **Figure 4e**). Finally, much poorer performance was achieved when a count based on only checkpoint module hits was used (**Figure 4f**). Our results also showed that the perturbation index is independent of the total number of gene hits for each compound and other parameters (see **Figure S2, S3, S4, S5, S6, S7, S8, S9**). In summary, the results demonstrate the effectiveness of the perturbation index, and confirm that the key factors which account for drug efficacy are primarily the hits on gatekeeper modules; and additionally, this could be further influenced by the ‘active’ control scope of the gatekeeper modules.

### Rationale and synergy quantification of drug combination

**Figure 5:**
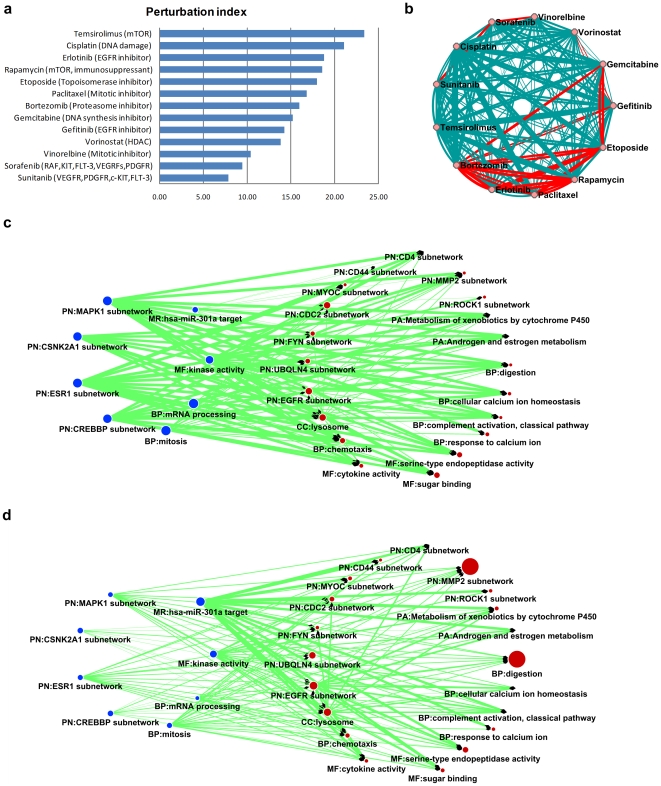
A proof-of-principle demonstration for a drug combination study based on POA analysis. **a**. Rank of drugs and agents in clinical development for lung cancer (NSCLC) according to their perturbation index. Text after agent name indicates the mechanism of action. **b**. The perturbation index of pair-wise combination of NSCLC agents. The widths of links between two drugs are proportional to their combined ***PI*** index (see **Methods**), and red links indicates the potential benefit of the combination: ***PI*** (combination) > maximum (***PI*** (drug 1), ***PI*** (drug 2)). **c**. POA of gemcitabine, red (at right) circles represent all gatekeeper modules. For clarification, selected checkpoint modules (top number of gene hits) are shown as blue (at left) circles. The size of circle is proportional to the number of gene hits for each module and the link widths are also proportional to the number of gene hits from the source node (checkpoint modules). **d**. POA of bortezomib with same schema. Gene module names start with a 2-character header indicating the gene module definition source, PN: protein subnetwork; PA: pathway; BP: Gene Ontology biological process; MF: Gene Ontology molecular function; CC: Gene Ontology cellular component; MR: microRNA targets.

Having established the validity of the perturbation index we then estimated it for lung cancer (NSCLC) drugs and related targeted agents in clinical development (**Figure 5a**). The first line treatment drug Cisplatin achieved a rank of two (***PI*** = 21.09, see **Figure 2a** for the Pattern of Action for Cisplatin). In the simulation of two-agent combinations, Bortezomib, the proteosome inhibitior, gained the largest number of benefits when combined with other agents (Erlotinib, Paclitaxel, Rapamycin, Etoposide, Gefitinib and Gemcitabine, **Figure 5b**), suggesting a multifaceted potential in combinatory treatment.

As a successful drug for treating multiple myeloma, Bortezomib is also being studied in the treatment of other types of cancer (There are 189 Bortezomib related clinical trials to date according to the NCI website: www.cancer.gov). The interference with ubiquitin pathways, which labels proteins for degradation by the proteasome, has proved to be a valid strategy for the development of anticancer drugs [30]. In a RNA interference (RNAi)-based synthetic lethal screen seeking paclitaxel chemosensitizer genes in a human NSCLC cell line, proteasome is the most enriched gene group [12]. Recently, a phase II clinical trial reported notable survival benefits in lung cancer (NSCLC) patients using a Bortezomib plus Gemcitabine/Carboplatin combination as first-line treatment [31]. In line with the above result, here we identified the combinatory benefits of both the Bortezomib-Paclitaxel and Bortezomib-Gemcitabine combos (**Figure 5b**). Impressively, in the intrinsic inter-module network, the gene modules ‘UBQLN4 (ubiquilin 4) subnetwork’ shared synergy with 360 gene modules (**Figure 2b**).

Taking Bortezomib-Bemcitabine as an example, we further studied the mechanism of drug combination benefits. Compared to the chemotherapy agent Gemcitabine (**Figure 5c**), the Pattern of Action for Bortezomib shows a more focused hit pattern (**Figure 5d**). For the gatekeeper module hit pattern, Bortezomib has relatively more hits on the ‘UBQLN4 (ubiquilin 4) subnetwork’, and shows a very strong association with the ‘MMP2 (matrix metallopeptidase) subnetwork’ and ‘digestion’, which are targeted less frequently by Gemcitabine. As matrix metallopeptidases play an important regulatory role in the ubiquitylation pathway [32], the synergistic benefit of the Bortezomib-Gemcitabine combo in bladder tumors is related to matrix metalloproteinases and other microenvironment factors [33]. In terms of checkpoint modules, Bortezomib also has more gene hits on microRNA target modules *has-mir-301a*, which is revealed as a human embryonic stem cell-specific microRNA [34].

The results for our initial design for the mechanism of drug combination synergy (**Figure 1e**) confirmed the proposed rationale: Gemcitabine serves as a drug establishing a baseline perturbation on the inter-module network, but Bortezomib could add a more focused perturbation on key gatekeeper modules which are linked to the checkpoint perturbation established by Gemcitabine (**Figure 5d**). Knowledge of a drug's mechanism of action is critical for successful optimization of therapeutic drugs, especially for rational design of drug combinations. Our models could serve as a powerful tool for generating testable hypotheses on the mechanism of synergistic drug combinations. For example, our result suggests that the MMP2 subnetwork might be one of the key gene modules which are involved in the synergy between Gemcitabine and Bortezomib (**Figure 5d**). If this hypothesis could be experimentally verified, a series of new drug combinations could be proposed based on this assumption.

## Discussion

The preclinical development process has been criticized for its inability to identify drugs that are most likely to succeed in the human clinic. Many attempts have been made to address this issue by creating novel genetically engineered animal models for human cancers [35]. However, creating novel animal models to mirror the natural distribution of mutations is still a challenge, due in part to heterogeneity and unknown mutations (i.e., structure aberrations), which need to be revealed via ongoing efforts such as next generation sequencing. In this context, *in silico* modeling or simulations, which are based on the heterogeneous patient populations, provide an alternative yet cost-effective way to identify key factors affecting success rate in the human clinic. The modern drug discovery and development process is mainly a forward (and stepwise) approach: from drug target identification, preclinical assessment and mechanism studies, towards clinical trials. The *in silico* model we present here establishes a new information link between clinical trials to aid informed preclinical decisions.

Our analysis scheme has several unique characteristics as a preclinical *in silico* modeling tool. Specifically these are: (1) mirroring drug behavior on heterogeneous patient populations; (2) cost-effectiveness: One of the key inputs for effective modeling is the prognosis data, which is already available for large populations in various cancer types. Furthermore, this kind of retrospective study is cheap and less time consuming; (3) flexibility: It is easy to integrate the model with compound action mechanisms or patterns such as, for example, the NCI 60 in vitro cell line screening data used in this study. (4) extensibility: The pool of gene modules serves as a ‘library of mechanisms’ to probe the intrinsic gene network, and the power of the model can be sustainably improved along with emerging new gene module definitions. The ongoing efforts on interrogating genetic and epigenetic functional elements (e.g., the ENCODE project [36]) will greatly enhance the available options for gene modules definition and improve the resolution, specificity and multi-faced coverage of biological processes. For example, our analysis shows that microRNA regulated genes are very informative data sources in terms of gene module definitions.

The view that genomic instability is the key factor in tumorigenesis and tumor progression has been the prevailing paradigm for many years. Based on this, most of modern oncology drug discovery efforts are targeting to the etiology of cancer by seeking the key genetic lesions which are driving the tumorigenesis. However, recent evidence suggests epigenetic plasticity is an alternative driving force for the somatic evolution of tumors [37], [38], and some novel therapeutic strategies such as epigenetic treatments have emerged [39]. Our results highlight that drug metabolism, microenvironment and immune system modulation play a pivotal role in determination of the robustness of cancer phenotype, and these modules have high epigenetic instability in tumor cells. Given the high connectivity of these gatekeeper modules, it is a reasonable inference that tumor cells could exploit the epigenetic plasticity within these key modules and thus gain a drug resistance phenotype, as suggested by the ‘phenotypic plasticity’ hypothesis [26] and the ‘epigenetic progenitor model’ of cancer [25].

The potential strategy that tumors could exploit against the drug treatment cannot be fully determined by etiology studies, but *in silico* systems biology modeling will provide a way to predict the survival strategies of a tumor when undergoing drug treatment. The task presented here mainly aims to identify the central players in the determination of the robustness of a cancer network, which is only the first step in using systems biology modeling in the battle against cancer. The next step will be behavior simulation based on this network. We believe that the next generation therapeutics might represent a paradigm shift from ‘etiology-based strategy’ towards ‘prediction-based strategy’ against the tumor. The former paradigm relies on the comprehensive understanding of tumor history, but the latter requires precise prediction of the tumor survival strategy under therapeutic interventions. Systems biology modeling such as we have presented in this study will enable this paradigm shift and make a unique contribution to this continually evolving challenge.

## Methods

### Construction of gene modules

A gene module was defined as a group of genes which share a similar function or regulation mechanism. The following types of information were used to construct gene modules: (1) Protein sub-network Data. In a protein-protein interaction network, nodes represent proteins and edges represent a physical protein interaction. A protein sub-network was defined by querying the nearest neighborhood nodes of high connectivity nodes (hubs, degree> = 20), and named according to the gene name of the hub protein. The human protein-protein interaction dataset in the HPRD (human protein reference database, www.hprd.org, Sep 1, 2007 release) was used as the source dataset. (2) Gene sets which share a common functionality in the gene annotation database. Here all three categories in the Gene Ontology were used: Biological Process, Molecular Function and Cellular Component (geneontology.org). The Entrez Gene ID to Gene Ontology mapping was downloaded from http://www.biomart.org. All genes associated with one GO term was defined as one gene module and the module was named according to the name/title of GO terms. (3) Pathway Data. Genes in one KEGG pathway (www.genome.jp/kegg) formed a gene module. (4) Protein complex data. Genes in one protein complex formed a gene module. The CORUM database [40](http://mips.gsf.de/genre/proj/corum/index.html) was used as the source dataset. (5) MicroRNA data. Genes regulated by the same microRNA formed one gene module (where predicted target genes of the microRNAs were taken from miRBase, http://www.ebi.ac.uk/enright-srv/microcosm/htdocs/targets/v5/, target gene set version 5). Because of the hierarchical structure of the ontology tree, the parent nodes (gene modules) in ontology hierarchy might inherit SINs from their children nodes (gene modules). To ensure the specificity of inter-module interaction, we control the gene module size and only gene modules containing between 100 and 200 genes were selected.

### Generation of compound sensitivity gene signatures

Biological response and gene expression data from the NCI/NIH Developmental Therapeutics Program In Vitro Cell Line Screening Project [20] (http://dtp.nci.nih.gov) was used to determine gene signatures for a series of compounds. The project screens test compounds against a panel of 60 cell lines and for each compound measures: (i) a biological response pattern (i.e., the GI50 value, the compound concentration that causes 50% cell growth inhibition) which is represented by a Response matrix R (compounds × cell lines); and (ii) the baseline gene expression profile for each compound for each of the 60 cell lines which is represented by a gene expression matrix G (genes × cell lines). For each compound, the Pearson Correlation Coefficients (PCC) between the GI50 pattern across 60 cell lines and each gene expression pattern across 60 cell lines were calculated [21], and genes with a PCC P-value<0.05 were selected as the compound sensitivity associated genes. The effects of other p-values were also examined but were not found to have much effect on the results (**Table S1**).

### Construction of inter-module cooperation networks from prognosis data

(1) Identification of query modules. Over-represented gene modules in genes interrogated in the NCI 60 project (gene expression matrix G) were detected by a fitting to a hypergeometric distribution (see **Supporting Text S1** for details). These identified modules were then used as query modules (**Figure 1c** **, A1;** **Figure 1e** **, A1**) to search for cooperative modules to form a directed network where all edges ran from the Query nodes to the Cooperative nodes;

(2) Identification of cooperative modules and creation of Disease-specific inter-module cooperation network. Cooperative modules were identified from prognosis data, which comprised microarray gene expression data generated from cancer patients and a prognosis that was classified as either good outcome (longer survival time) or bad outcome (shorter survival time). Data sets were analyzed for lung cancer [41], breast cancer [42], ovarian cancer [43] and leukemia (AML) [44] (**Supporting Text S1**). For each module in the query set from (1), we scanned the prognosis data to identify synergistic gene partner, resulting in a synergistic gene list. Synergistic partners were identified using an information theoretic measure of synergy based on the patient microarray expression data and the prognosis outcome [45] (**Supporting Text S1**). Over-represented gene modules in this synergy gene list were identified by fitting to a hypergeometric distribution, resulting a list of gene modules (**Figure 1c** **, B1;** **Figure 1e** **, B1**). For each cancer dataset, this produced a Disease-specific Inter-Module Cooperation Network (IMCN) consisting of Query and Cooperative nodes with edges running from Query nodes to Cooperative nodes (**Figure 1e**).

(3) Perturbation modules and Pattern of Action (POA): In order to investigate the effect of a drug compound on a IMCN we generated an associated Pattern of Action (POA) for each compound. This was done by selecting modules identified in the previous step that were associated with the compound and overlaying them on the disease-specific IMCN (**Figure 1f**).

### Compound Pattern of Action map and the definition of perturbation index

To quantify the compound ‘Pattern of Action’ we defined a perturbation index that was defined in terms of the number of compound gene ‘hits’ on modules within the IMCN. Compound gene ‘hits’ were defined as the genes which were significantly correlated with compound sensitivity across 60 cell lines. If at least one gene within a module (node in IMCN) is hit by the compound, this module is said to be hit by the compound. The perturbation index (*PI*) of a compound *c* was defined as
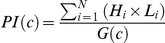
where *N* is the number of gatekeeper modules. For each gatekeeper module, *H*_i_ is the number of gene hits by compound *c* and *L*_i_ is the number of active links, where a link is formed when both source node and target node are hit by compound *c*. The index is normalized by the total number of gene hits by the compound: *G(c)*.

### Bootstrapping-based assessment of ability of perturbation index to discriminate successful drugs from the candidate pool

To investigate whether the Perturbation Index could be used to identify successful drugs from a broader range of compounds, a Drug Candidate set ***C*** was downloaded from the NCI Standard Agent Database (http://www.dtp.nci.nih.gov/docs/cancer/searches/standard_agent.html); a set ***S*** of successful lung cancer drugs were identified from a review paper [46], and overlapped with ***C***. The perturbation index was then calculated for each entry in ***C*** and ***S***. To test the significance of the differences in the PI distributions for **S** and **C** we utilized a bootstrap based method (with replacement). Given there are *n* compounds in **S**, we generated the background distribution by sampling *n* compounds 20000 times from candidate pool **C**, and calculated the average of the perturbation index. Then we calculated the p-value based on the number of times the average index of the candidate pool was larger than the index for set **S**.

### Quantification of drug combination synergy

To examine the effects of combined drug treatment, the drug list was expand to include both approved lung cancer drugs (set ***S*** **,** from review paper [46]) and new molecularly targeted drugs in clinical development (from review paper [47]), and overlapped with screened compounds in NCI 60 cell lines screening project**.** All possible pairwise combinations of compounds in this combined list were investigated. For each combination of two compounds, the union of sensitivity associated gene lists of the two compounds was formed and the perturbation index of each drug combination was calculated in the same way as the individual compound.

### Determination of genetic and epigenetic aberration frequency in inter-module network

It has been proposed that events such as DNA mutation and CpG methylation may play an important role in cancer. Thus, genes that were highly represented in the IMCNs were identified and then inspected to see their frequency characteristics of mutation and methylation events.

(1) Identification of highly represented genes in gatekeeper and checkpoint modules

As the gene modules were created from relationships defined in ontologies, protein interaction networks, pathways and miRNA targets, individual genes will, in general, be present in multiple gene modules. Therefore, the number of times each gene appeared in checkpoint modules and gatekeeper modules, respectively, was calculated and two sets of the 10% and 20% most frequently occurring genes were selected as representative genes. This produced 1230 representative genes for checkpoint modules and 183 genes for gatekeeper modules for a 10% cutoff; and 2461 genes for checkpoint modules, 366 genes for gatekeeper modules for a 20% cutoff.

(2) Gene-level somatic mutation and DNA methylation data

Somatic mutation data was obtained from the Sanger Institute Catalogue Of Somatic Mutations In Cancer web site, http://www.sanger.ac.uk/cosmic (version 42, May 28, 2009) [48]. Aberrant CpG methylation data in human tumours was obtained from the ‘MethCancer DB’ web site, http://www.methcancerdb.net (April 22, 2008) [49].

(3) Somatic mutation and DNA methylation incident rates

Incident rate of somatic mutations for each type of gene module (gatekeeper and checkpoint) was defined as:
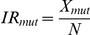



Where *X_mut_* is the number of mutated representative genes, and *N* is the total number of representative genes.

Incident rate of aberrant CpG methylation (*IR_met_*) for each type of gene module was defined as:
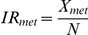



Where *X_met_* is the number of aberrantly methylated representative genes, and *N* is the total number of representative genes.

To test whether the gatekeeper *IR_mut_* was significantly different from the checkpoint *IR_mut_*, a p-value was calculated according to 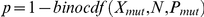



Where *binocdf* is the Binomial cumulative distribution function, *X_mut_* is the number of mutated gatekeeper genes in the test set, *N* is the total number of gatekeeper genes in the test set and *P_mut_* is the probability of a mutation event in checkpoint modules (equal to the checkpoint *IR_mut_*).

The final two-sided p-value *p_2_* was then calculated from
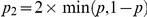
where *min* returns the smaller of *p* or *1-p*.

The p-value for methylation events in the gatekeeper and checkpoint modules was calculated in the same manner.

## Supporting Information

Text S1 Supplementary methods.
(0.12 MB DOC)

Table S1 The effect of drug-gene association P-value cutoff on bootstrap result.
(0.04 MB DOC)

Figure S1 Gatekeeper gene modules in various type of cancer.
(0.63 MB TIF)

Figure S2 Bootstrap results for pseudo index. To test whether our results are biased by study bias introduced by gene module definition, we defined a pseudo index for each compound  =  Nnet/Ntotal, where Nnet is the number of gene hits in lung cancer network for a given compound, and Ntotal is the number of genes in the compound sensitivity-associated gene list. The same bootstrap procedure (as demonstrated in Figure 4) run on this pseudo index and result are demonstrated here. Blue line shows background distribution and the red line shows the average pseudo index of successful drugs. This figure clearly shows that this pseudo index could not discriminate success drugs from candidate pool (P-value>0.05).
(0.45 MB TIF)

Figure S3 The effect of network size on perturbation index. This figure shows that the number of network hits is proportional to the overall number of compounds gene signatures (left), whereas the perturbation index is independent of the number of compounds gene signatures (right).
(0.56 MB TIF)

Figure S4 The effect of gene module size on bootstrap results (gene module size from 50–100 genes/modules, 24 gatekeeper modules). In the main text we only selected modules which contained 100–200 genes. To test whether our results were sensitive to gene module size, we investigated the bootstrap results (Figure 4c–4f in main text) when we changed the gene module size. We investigated ranges 50–100, 50–200, 50–300, 50–400, 50–500, 50–600 (Figures S4–S9). In line with result demonstrated in Figure 4c–4f, the bootstrapped P-values of the perturbation index (top left plot in each figure) are always smaller (better) than the modified perturbation index definition (as control, bottom left of each plot).This plot shows bootstrap results for evaluating if the average Perturbation index (PI) of successful drugs against lung cancer (NSCLC) is significantly different from the candidate compounds (see Methods). The meaning of each subplot is exactly the same with Figure 4c–4f. Blue line shows background distribution and the red line shows the average PI of successful drugs. Top-left: the bootstrap result by using defined PI. We also considered modified PI definitions and investigated their effect/contribution on the performance of PI. These modifications include: top-right: result from pseudo PI definition by using checkpoint modules information to replace gatekeeper modules information, bottom-left: result from pseudo PI definition by using gatekeeper modules hits; bottom-right: result from pseudo PI definition by using checkpoint modules hits.
(0.75 MB TIF)

Figure S5 The effect of gene module size on bootstrap results (gene module size from 50–200 genes/modules, 44 gatekeeper modules). See Figure S4 for details.
(0.72 MB TIF)

Figure S6 The effect of gene module size on bootstrap results (gene module size from 50–300 genes/modules, 57 gatekeeper modules). See Figure S4 for details.
(0.71 MB TIF)

Figure S7 The effect of gene module size on bootstrap results (gene module size from 50–400 genes/modules, 60 gatekeeper modules). See Figure S4 for details.
(0.71 MB TIF)

Figure S8 The effect of gene module size on bootstrap results (gene module size from 50–500 genes/modules, 64 gatekeeper modules). See Figure S4 for details.
(0.71 MB TIF)

Figure S9 The effect of gene module size on bootstrap results (gene module size from 50–600 genes/modules, 72 gatekeeper modules). See Figure S4 for details.
(0.71 MB TIF)
