# Supplementary material for: Pre-Clinical Drug Prioritization via Prognosis-Guided Genetic Interaction Networks
Source: PLoS One. 2010 Nov 10;5(11):e13937. doi: 10.1371/journal.pone.0013937 (PMC2978107; doi:10.1371/journal.pone.0013937)
Supplement: Text S1 — Supplementary methods. (0.12 MB DOC) [file pone.0013937.s001.doc]

## Supplementary methods

### Detecting over-represented (enriched) gene modules in a gene set by hypergeometric distribution

Detecting the enriched gene modules or gene functional category (i.e., Gene Ontology) is a common bioinformatics task. The term ‘enrichment’ means that the distribution density of input gene set in given gene modules is significantly higher than the reference model. We adapted the common used hypergeometric distribution method. That is, the cumulative probability of having up to x overlapped genes when comparing the query gene list (K genes) to each candidate gene modules (N genes) was calculated by:

Where M is the size of the whole gene set (i.e., the number of all human genes).

### Identifying the Synergistically Inferred Nexus (SIN)

For each gene **G_1_** in a given gene modules, we scan the candidate gene space (all genes in the prognosis data set, that is, all Entrez gene IDs corresponding to each probe set in the Affymetrix U133A microarray, and filtered by variance of cross patient samples), and calculate the pair-wise **synergy index** of **G_1_** with candidate gene **G_2_** following[1]:

In this formula, *G_1_* and *G_2_* represent the expression values of two genes, and *C* represents the phenotype (here, the prognosis outcome. For example, a two-state vector which 1 means “bad outcome” or “dead”, 0 means “good outcome” or “survival”), I(X;Y) represents mutual information between two random variables (i.e., the expression levels of *G_1_* and *G_2_*), .

The above formula is equivalent to:

And the mutual information of two random variables is calculated by:

For each gene, we transform the expression value into two states (*high* and *low*) by using median value across the whole patient set as the cutoff. Mutual information is calculated via the method and toolbox proposed by Peng [2].

In this pilot study of the proposed framework, we checked the distribution of *synergy index*, and used a threshold (0.02) of *synergy index* to select the synergistic gene pairs.

### The prognosis data set used in the present study

1. Lung cancer (NSCLC) [4], the Gene Expression Omnibus (GEO) accession no. [GSE3593](http://www.ncbi.nlm.nih.gov/geo/query/acc.cgi?acc=GSE3593);
2. Breast cancer [5]; the Gene Expression Omnibus (GEO) accession no. [GSE2034](http://www.ncbi.nlm.nih.gov/projects/geo/query/acc.cgi?acc=GSE2034);
3. Ovarian cancer [6]; the Gene Expression Omnibus (GEO) accession no. [GSE3149](http://www.ncbi.nlm.nih.gov/geo/query/acc.cgi?acc=GSE3149);
4. AML [7]; the Gene Expression Omnibus (GEO) accession no. [GSE12417](http://www.ncbi.nlm.nih.gov/geo/query/acc.cgi?acc=GSE12417).

## References

**1. Anastassiou, D., *Computational analysis of the synergy among multiple interacting genes.* Mol Syst Biol, 2007. 3: p. 83.**

**2. Peng, H., F. Long, and C. Ding, *Feature selection based on mutual information: criteria of max-dependency, max-relevance, and min-redundancy.* IEEE Trans Pattern Anal Mach Intell, 2005. 27(8): p. 1226-38.**

**3. Bamford, S., et al., *The COSMIC (Catalogue of Somatic Mutations in Cancer) database and website.* Br J Cancer, 2004. 91(2): p. 355-8.**

**4. Potti, A., et al., *A genomic strategy to refine prognosis in early-stage non-small-cell lung cancer.* N Engl J Med, 2006. 355(6): p. 570-80.**

**5. Wang, Y., et al., *Gene-expression profiles to predict distant metastasis of lymph-node-negative primary breast cancer.* Lancet, 2005. 365(9460): p. 671-9.**

**6. Bild, A.H., et al., *Oncogenic pathway signatures in human cancers as a guide to targeted therapies.* Nature, 2006. 439(7074): p. 353-7.**

**7. Metzeler, K.H., et al., *An 86-probe-set gene-expression signature predicts survival in cytogenetically normal acute myeloid leukemia.* Blood, 2008. 112(10): p. 4193-201.**
