# Supplementary material for: Pre-Clinical Drug Prioritization via Prognosis-Guided Genetic Interaction Networks
Source: PLoS One. 2010 Nov 10;5(11):e13937. doi: 10.1371/journal.pone.0013937 (PMC2978107; doi:10.1371/journal.pone.0013937)
Supplement: Table S1 — The effect of drug-gene association P-value cutoff on bootstrap result. (0.04 MB DOC) [file pone.0013937.s002.doc]

Table S1 The effect of drug-gene association P-value cutoff on bootstrap result

| Drug-gene association P-value cutoff | Bootstrap P-value for perturbation index in **Fig.4c** | Bootstrap P-value for **modified** perturbation index in **Fig.4e** |
| --- | --- | --- |
| 0.2 | 0.1763 | 0.2615 |
| 0.1 | 0.0146 | 0.0460 |
| 0.05 | 0.0042 | 0.0800 |

To test whether our perturbation result (**Fig.4**) is sensitive to the P-value cutoff which is used to generate drug-gene associations (see **Methods**: “For each compound, the Pearson Correlation Coefficients (PCC) between the GI50 pattern across 60 cell lines and each gene expression pattern across 60 cell lines were calculated, and genes with a PCC P-value<0.05 were selected as the compound sensitivity associated genes.”), we re-calculated the perturbation result (**Fig.4**) for various drug-gene association p-value cutoffs. It was found that the conclusion we made based on **Fig.4** is relatively consistent, the perturbation index (PI) bootstrap p-value is always smaller than the modified version of the PI (as the control), and is still significant when the drug-gene association P-value cutoff set to 0.1. The result of drug-gene association p-values less than 0.05 are not shown, because there are not enough gene hits for most gene modules when the P-value cutoff is too stringent.
