# Supplementary figures and images for: Pre-Clinical Drug Prioritization via Prognosis-Guided Genetic Interaction Networks
Source: PLoS One. 2010 Nov 10;5(11):e13937. doi: 10.1371/journal.pone.0013937 (PMC2978107; doi:10.1371/journal.pone.0013937)

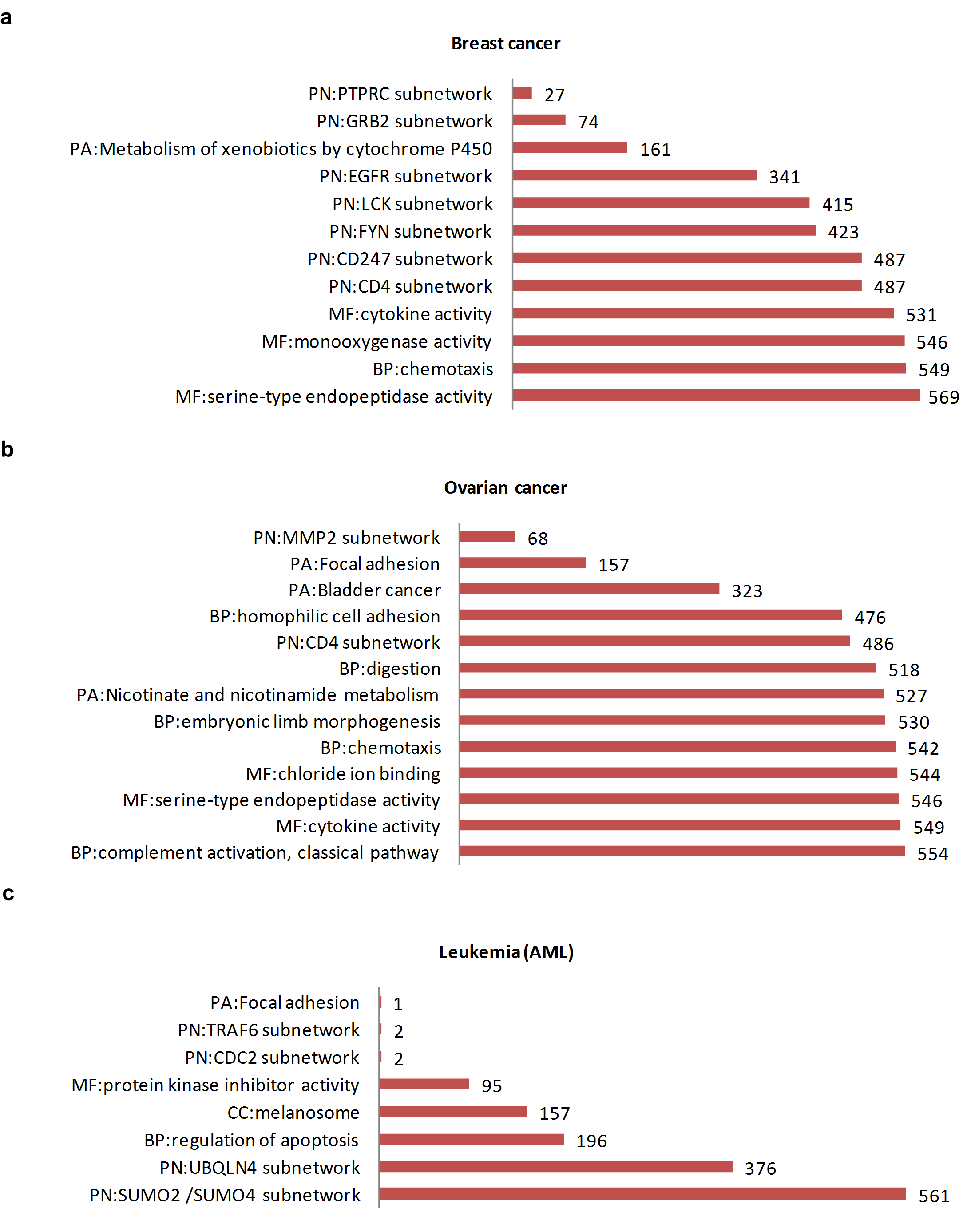

Supplement: Figure S1 — Gatekeeper gene modules in various type of cancer. (0.63 MB TIF) [file pone.0013937.s003.tif]

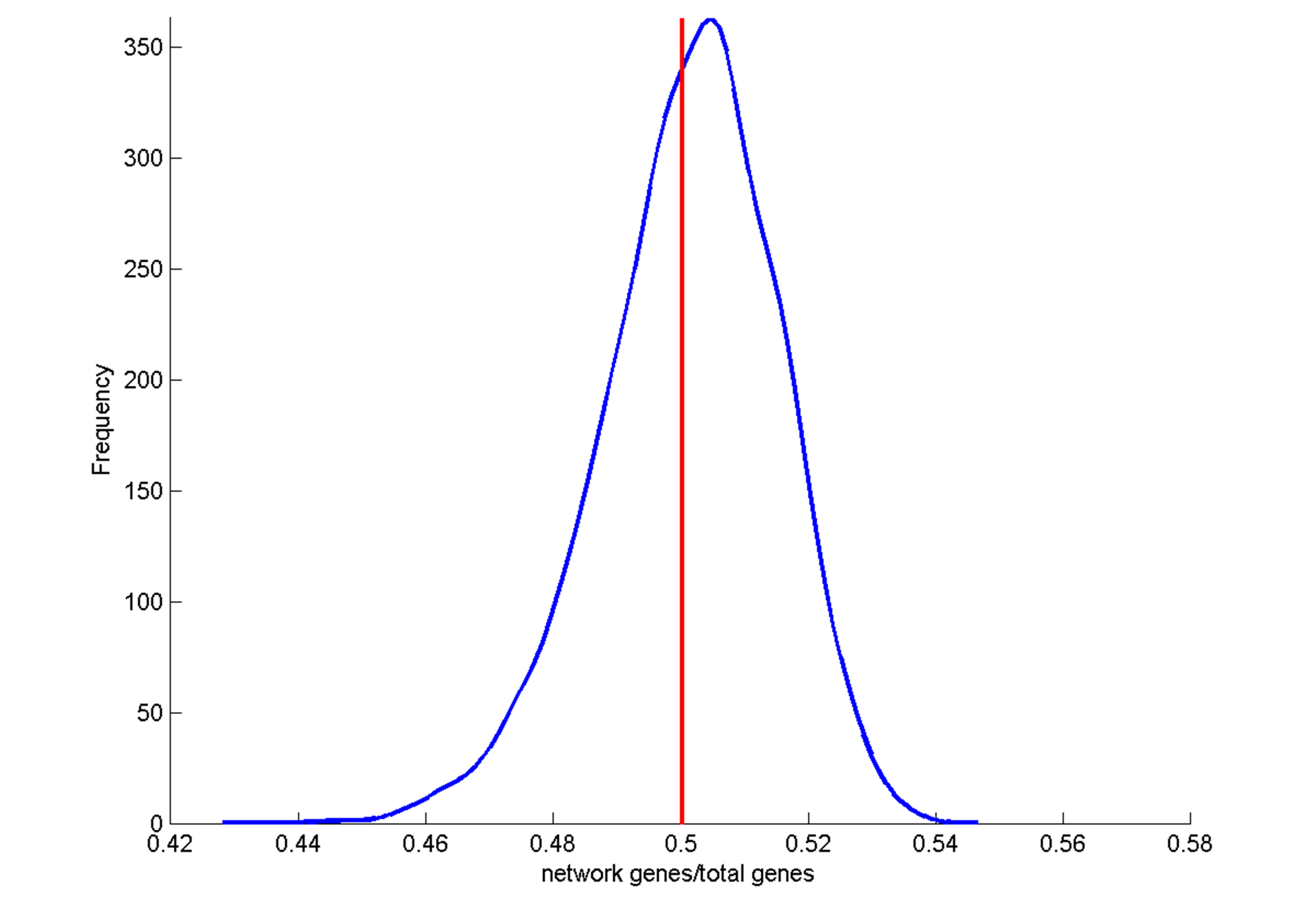

Supplement: Figure S2 — Bootstrap results for pseudo index. To test whether our results are biased by study bias introduced by gene module definition, we defined a pseudo index for each compound = Nnet/Ntotal, where Nnet is the number of gene hits in lung cancer network for a given compound, and Ntotal is the number of genes in the compound sensitivity-associated gene list. The same bootstrap procedure (as demonstrated in Figure 4) run on this pseudo index and result are demonstrated here. Blue line shows background distribution and the red line shows the average pseudo index of successful drugs. This figure clearly shows that this pseudo index could not discriminate success drugs from candidate pool (P-value>0.05). (0.45 MB TIF) [file pone.0013937.s004.tif]

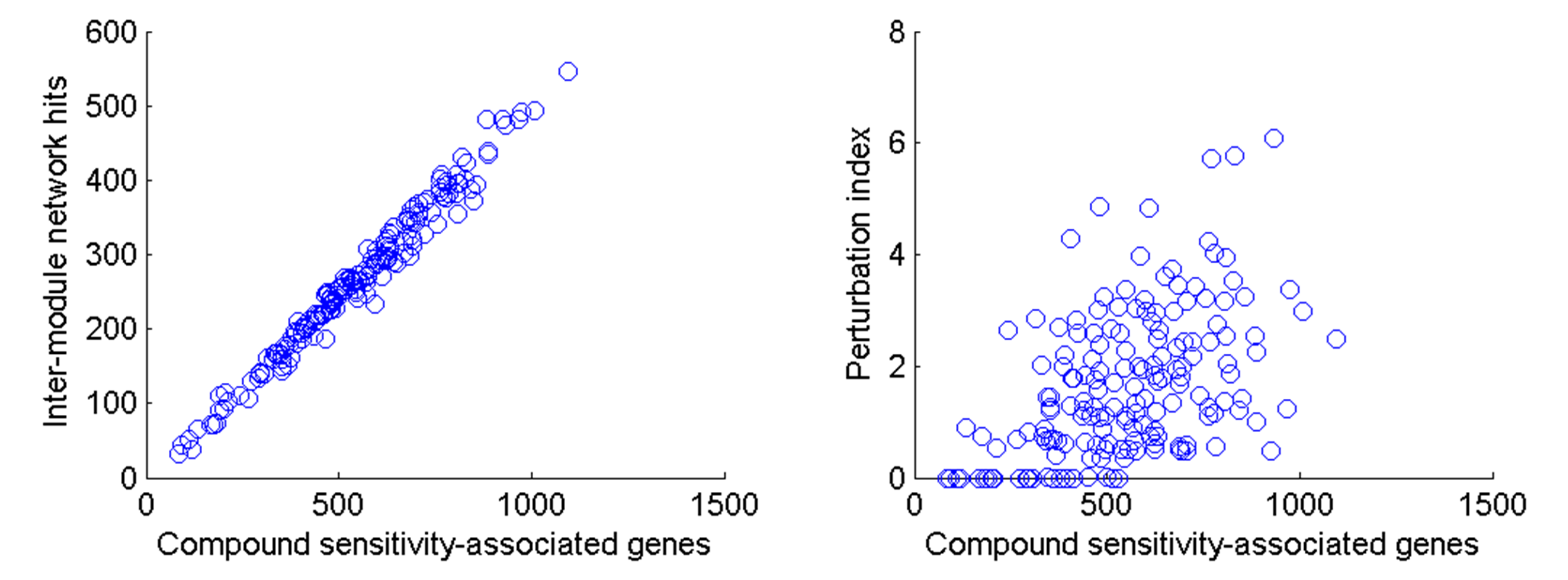

Supplement: Figure S3 — The effect of network size on perturbation index. This figure shows that the number of network hits is proportional to the overall number of compounds gene signatures (left), whereas the perturbation index is independent of the number of compounds gene signatures (right). (0.56 MB TIF) [file pone.0013937.s005.tif]

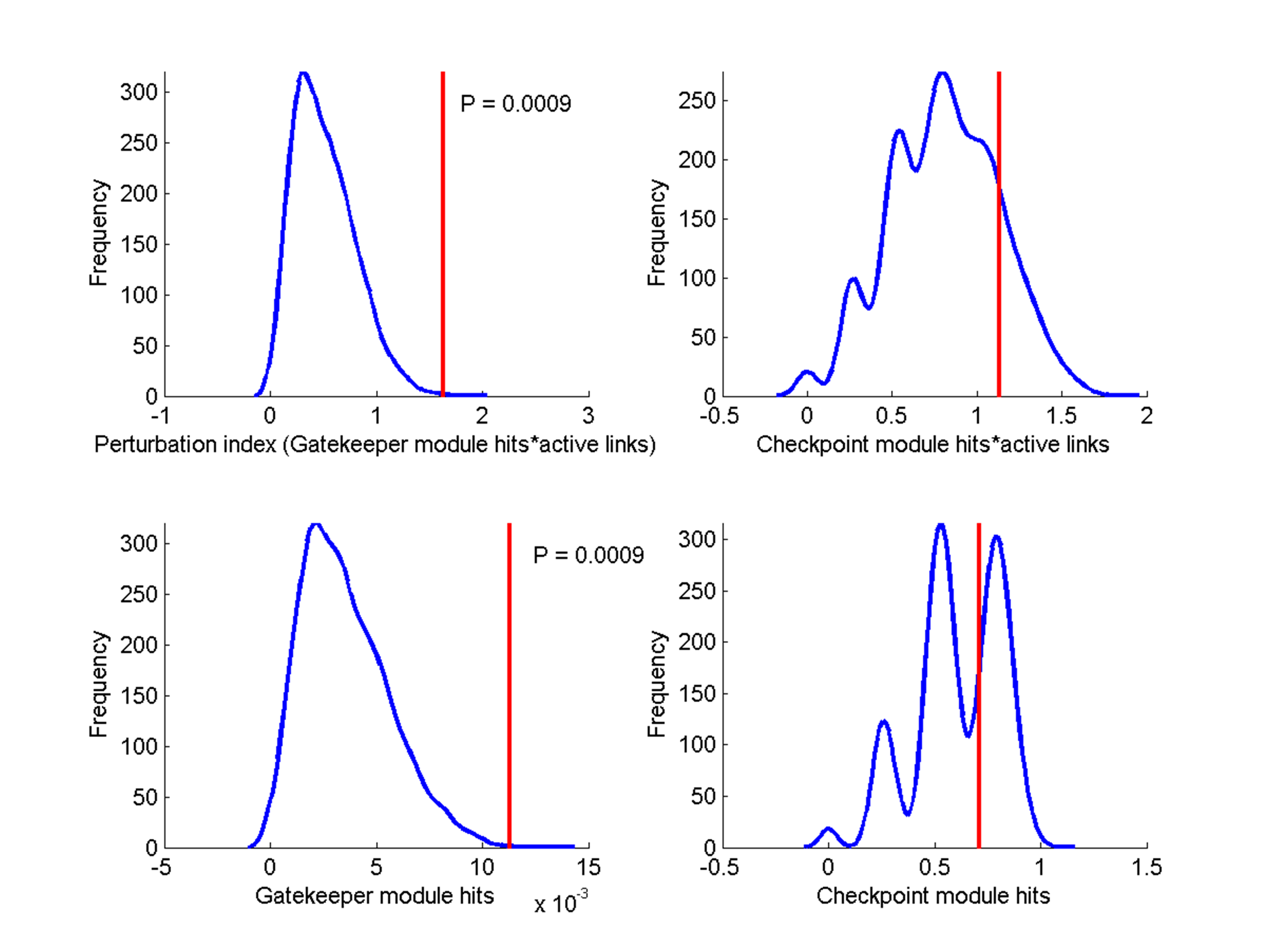

Supplement: Figure S4 — The effect of gene module size on bootstrap results (gene module size from 50–100 genes/modules, 24 gatekeeper modules). In the main text we only selected modules which contained 100–200 genes. To test whether our results were sensitive to gene module size, we investigated the bootstrap results (Figure 4c–4f in main text) when we changed the gene module size. We investigated ranges 50–100, 50–200, 50–300, 50–400, 50–500, 50–600 (Figures S4–S9). In line with result demonstrated in Figure 4c–4f, the bootstrapped P-values of the perturbation index (top left plot in each figure) are always smaller (better) than the modified perturbation index definition (as control, bottom left of each plot).This plot shows bootstrap results for evaluating if the average Perturbation index (PI) of successful drugs against lung cancer (NSCLC) is significantly different from the candidate compounds (see Methods). The meaning of each subplot is exactly the same with Figure 4c–4f. Blue line shows background distribution and the red line shows the average PI of successful drugs. Top-left: the bootstrap result by using defined PI. We also considered modified PI definitions and investigated their effect/contribution on the performance of PI. These modifications include: top-right: result from pseudo PI definition by using checkpoint modules information to replace gatekeeper modules information, bottom-left: result from pseudo PI definition by using gatekeeper modules hits; bottom-right: result from pseudo PI definition by using checkpoint modules hits. (0.75 MB TIF) [file pone.0013937.s006.tif]

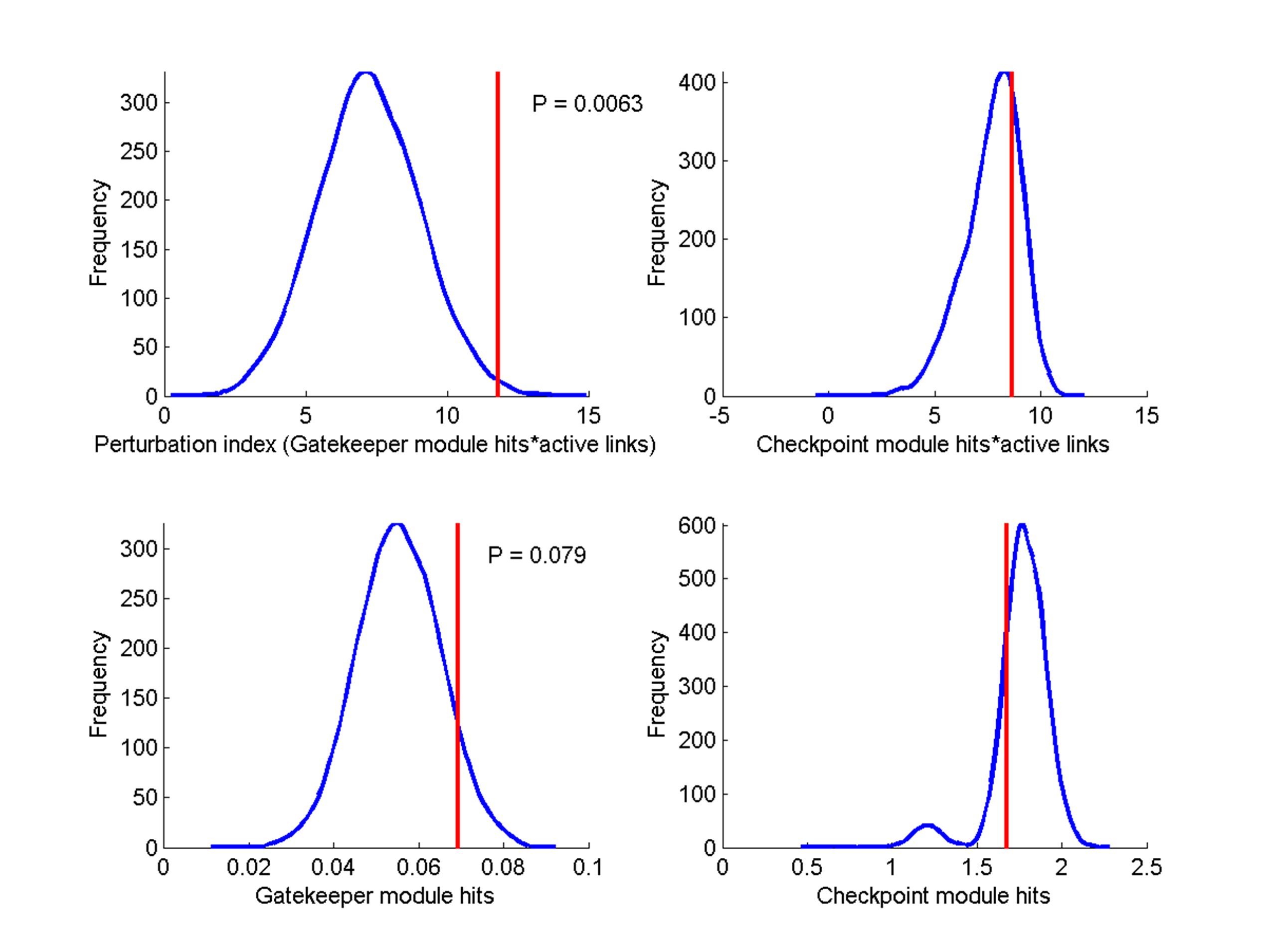

Supplement: Figure S5 — The effect of gene module size on bootstrap results (gene module size from 50–200 genes/modules, 44 gatekeeper modules). See Figure S4 for details. (0.72 MB TIF) [file pone.0013937.s007.tif]

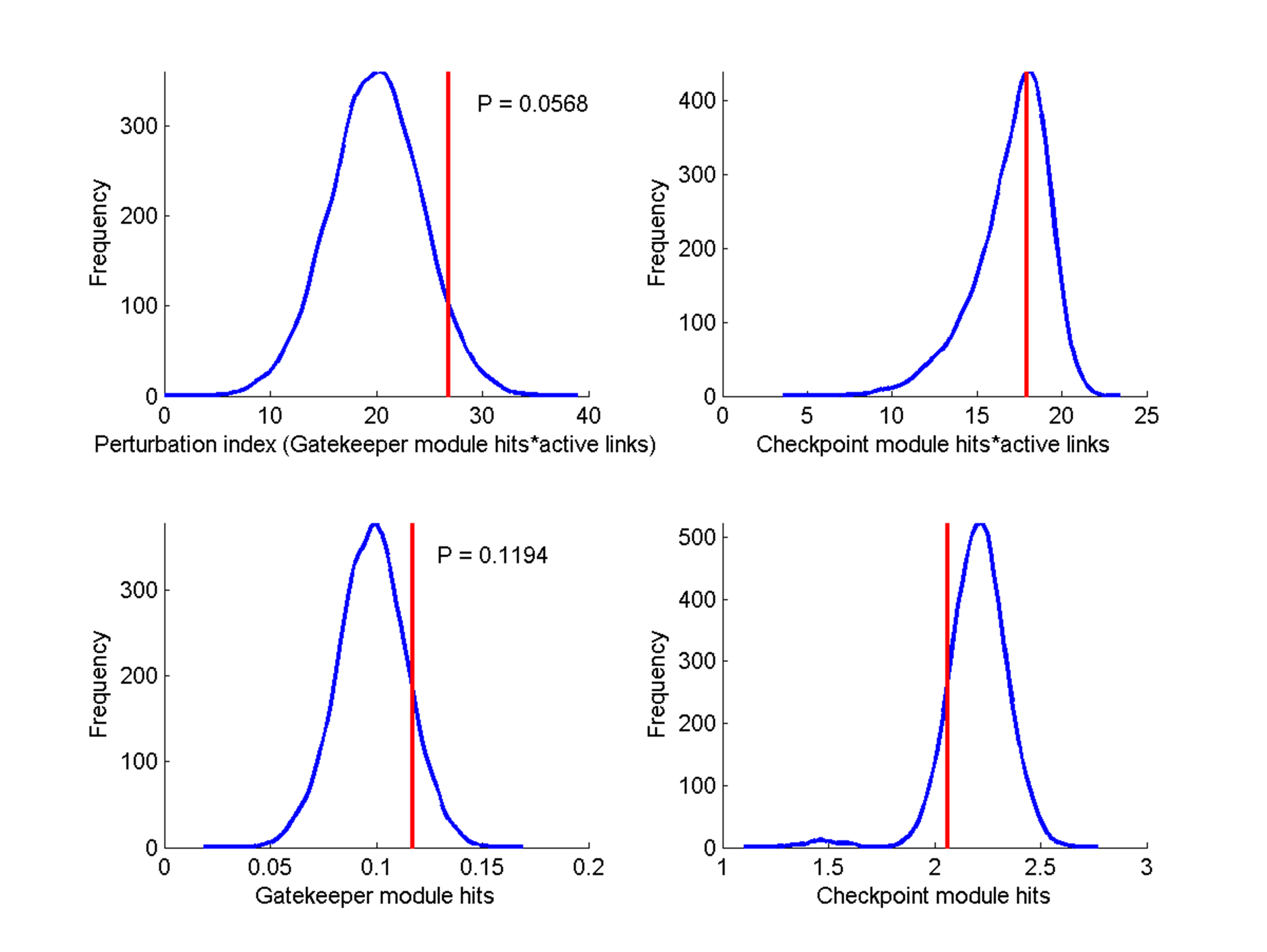

Supplement: Figure S6 — The effect of gene module size on bootstrap results (gene module size from 50–300 genes/modules, 57 gatekeeper modules). See Figure S4 for details. (0.71 MB TIF) [file pone.0013937.s008.tif]

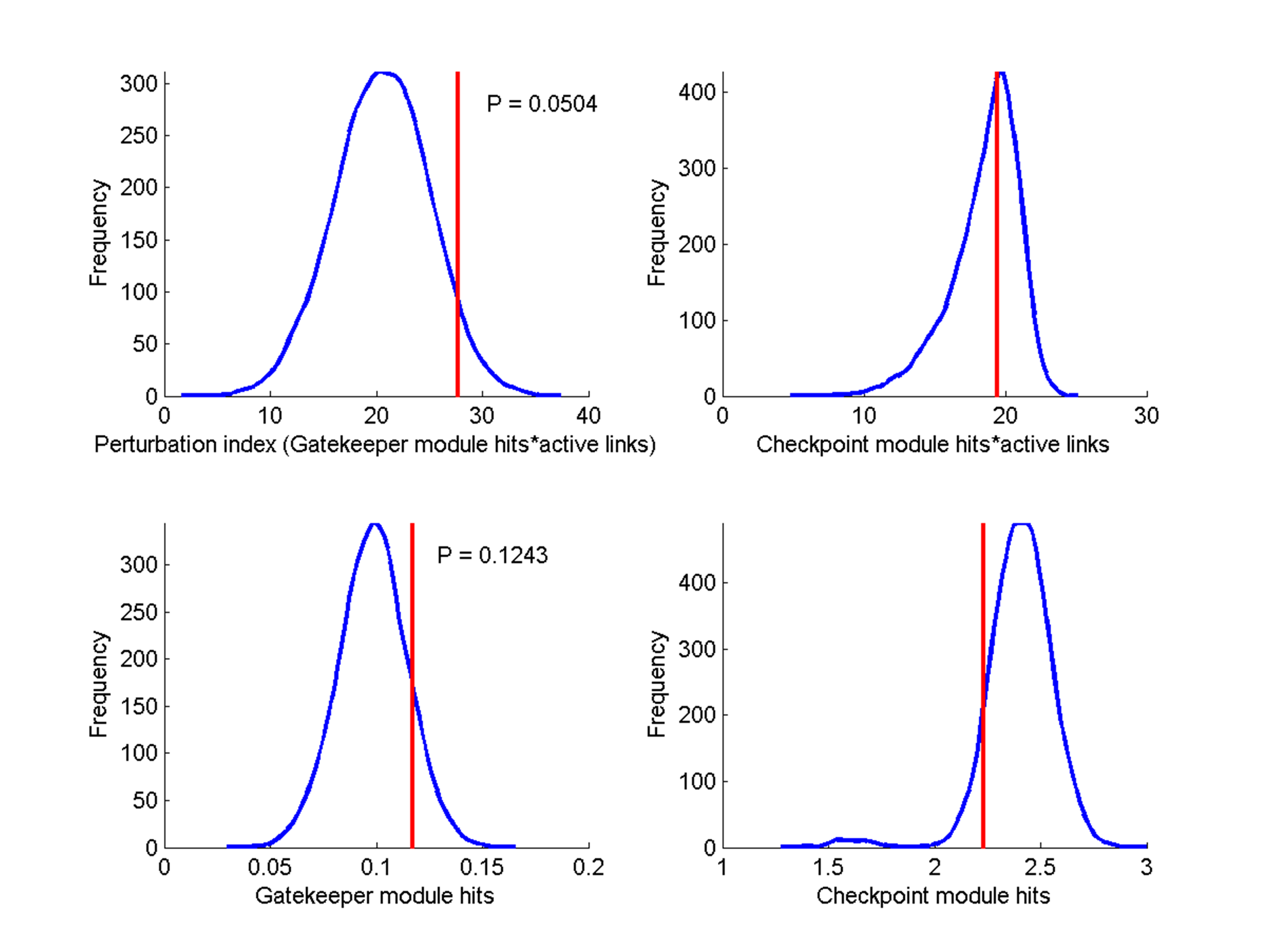

Supplement: Figure S7 — The effect of gene module size on bootstrap results (gene module size from 50–400 genes/modules, 60 gatekeeper modules). See Figure S4 for details. (0.71 MB TIF) [file pone.0013937.s009.tif]

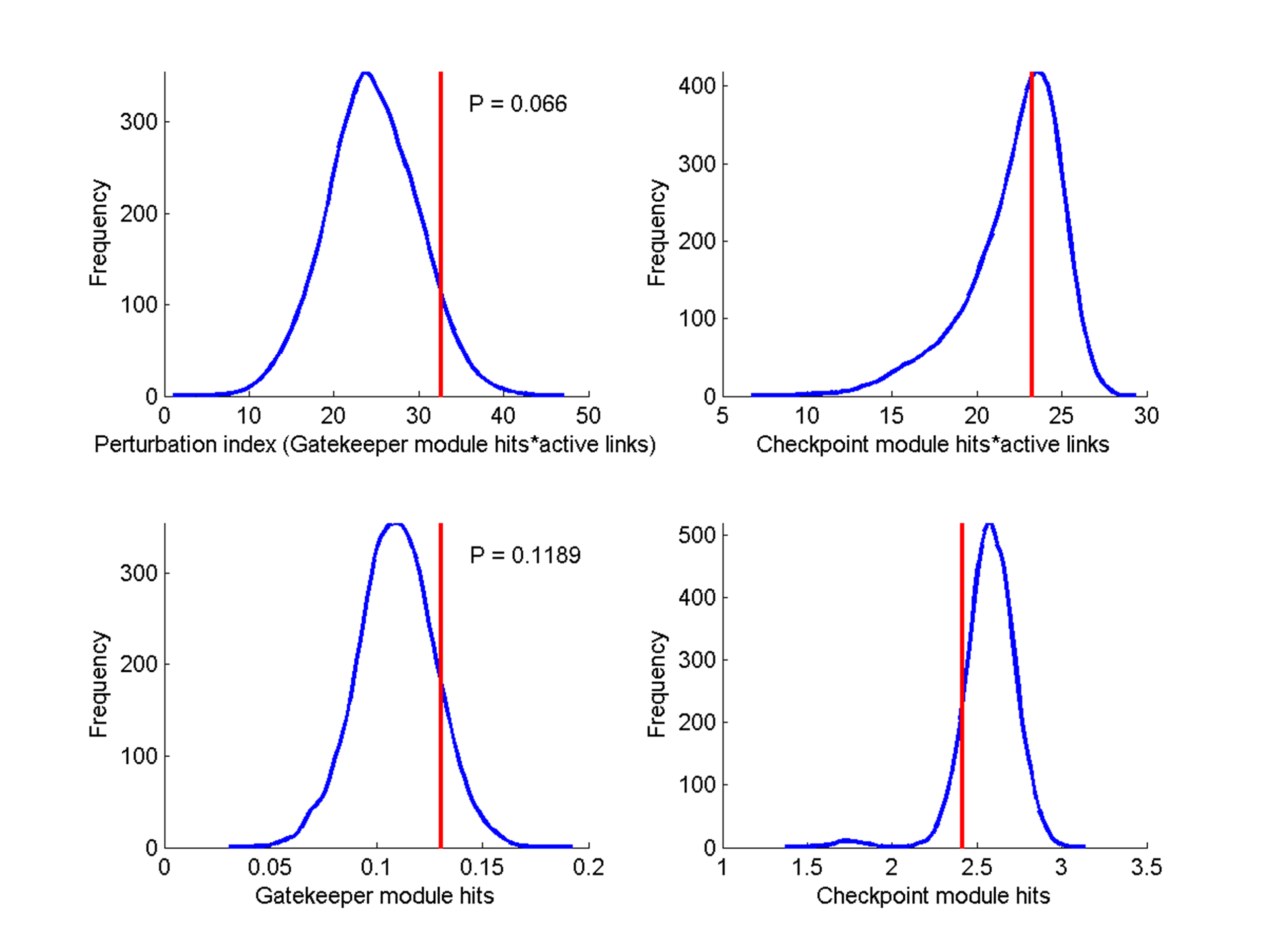

Supplement: Figure S8 — The effect of gene module size on bootstrap results (gene module size from 50–500 genes/modules, 64 gatekeeper modules). See Figure S4 for details. (0.71 MB TIF) [file pone.0013937.s010.tif]

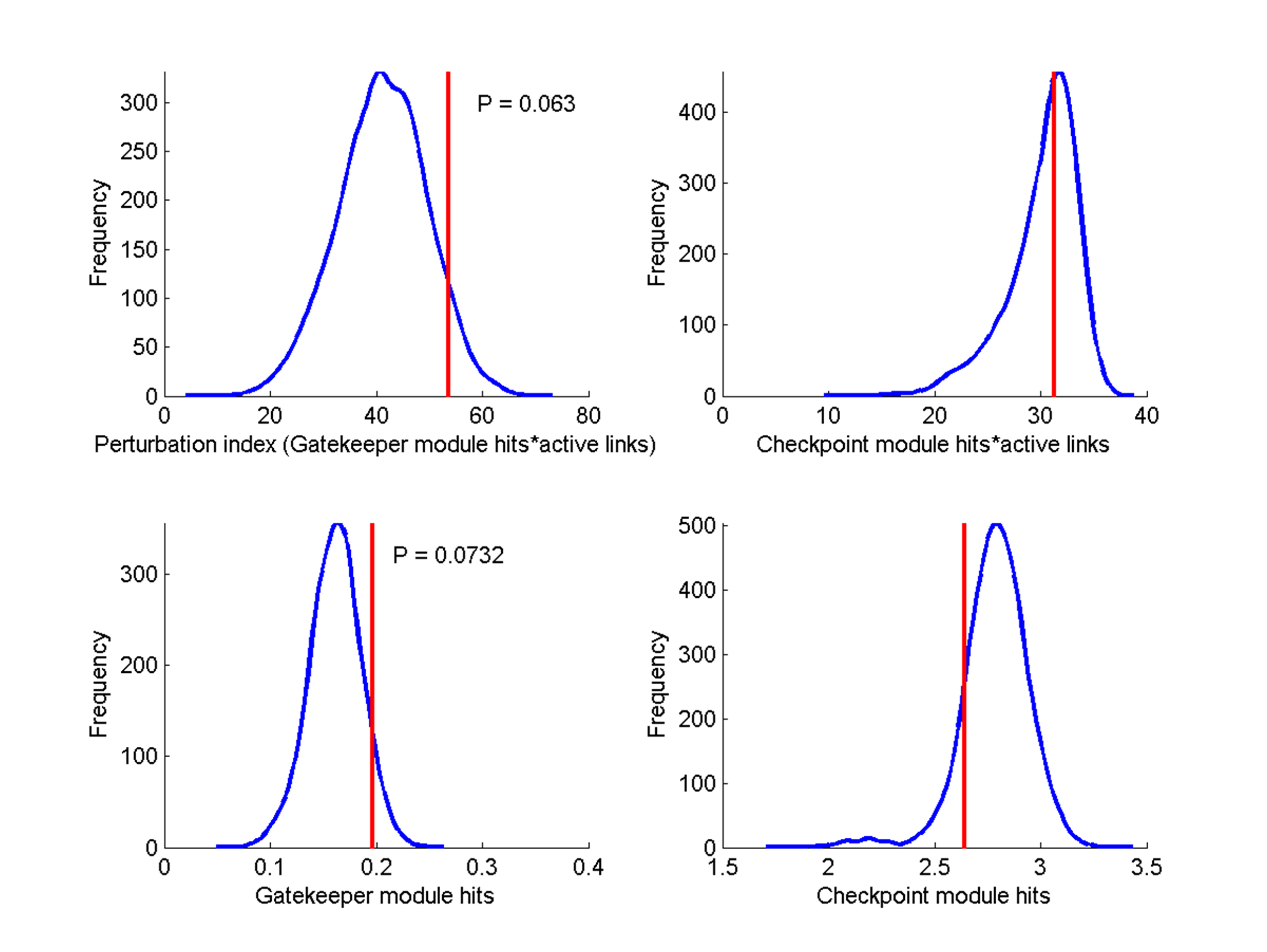

Supplement: Figure S9 — The effect of gene module size on bootstrap results (gene module size from 50–600 genes/modules, 72 gatekeeper modules). See Figure S4 for details. (0.71 MB TIF) [file pone.0013937.s011.tif]
